# Association of *APOEε4* with Centiloids in Hispanic and non‐Hispanic white cohorts

**DOI:** 10.1002/alz70856_098065

**Published:** 2025-12-24

**Authors:** Cally Xiao, Ganna Blazhenets, Renaud La Joie, Ioannis Pappas, Arthur W. Toga

**Affiliations:** ^1^ Laboratory of Neuro Imaging, Stevens Neuroimaging and Informatics Institute, Keck School of Medicine, University of Southern California, Los Angeles, CA, USA; ^2^ Memory and Aging Center, UCSF Weill Institute for Neurosciences, University of California, San Francisco, San Francisco, CA, USA

## Abstract

**Background:**

Our previous meta‐analysis reported that the Alzheimer's disease (AD) risk allele *APOEε4* was associated with fewer mild cognitive impairment (MCI) cases in Hispanic participants compared to non‐Hispanic white (NHW) participants. To further decipher the influence of *APOE* genotype on AD pathology, we are conducting a meta‐analysis to determine if there are differences between ethnicities in the association of *APOE* genotype with brain amyloid measured by positron emission tomography (PET) imaging and standardized to the Centiloid scale.

**Method:**

Source datasets discovered on the Global Alzheimer's Association Interactive Network (GAAIN) include the Anti‐Amyloid Treatment in Asymptomatic Alzheimer's (A4) study, the Alzheimer's Disease Neuroimaging Initiative (ADNI), the Health and Aging Brain Study: Health Disparities (HABS‐HD) cohort, the Imaging Dementia–Evidence for Amyloid Scanning (IDEAS) study, and the Standardized Centralized Alzheimer's and Related Dementias Neuroimaging (SCAN) study managed by the National Alzheimer's Coordinating Center (NACC). The combined dataset consisted of Hispanic or NHW participants who had Centiloids calculated from amyloid PET, which was reported by the individual studies, resulting in 16,831 participants (7.8% Hispanic).

**Result:**

The random effects meta‐analysis showed that Hispanic participants had significantly lower mean Centiloid values than NHW participants (*p* <0.001). A lower proportion of Hispanic participants were amyloid positive (32% vs. 52% in NHW), as defined by a Centiloid cutoff of 20. In only those who were amyloid‐positive, Hispanic participants diagnosed with dementia had significantly lower Centiloids than NHW participants diagnosed with dementia (*p* <0.001). In addition, in only those who were amyloid‐positive, there was no difference in Centiloids between ethnicities in the *APOEε4*‐negative cohort, but in the *APOEε4*‐positive cohort, Hispanic participants had slightly but significantly lower Centiloids than NHW participants (*p* = 0.05).

**Conclusion:**

Worsening cognitive diagnoses and the presence of the *APOEε4* allele were associated with higher Centiloids in both Hispanic and NHW cohorts, but Hispanic participants had significantly lower Centiloids than NHW participants at these stages. We plan to include additional datasets to further explore interactions between demographics, genotype, diagnosis, and Centiloids in Hispanic and NHW cohorts.

